# Potential bioactive compounds and mechanisms of *Fibraurea recisa Pierre* for the treatment of Alzheimer’s disease analyzed by network pharmacology and molecular docking prediction

**DOI:** 10.3389/fnagi.2022.1052249

**Published:** 2022-12-08

**Authors:** Shishuai Wang, Yixuan Ma, Yuping Huang, Yuhui Hu, Yushan Huang, Yi Wu

**Affiliations:** ^1^Key Laboratory of Prevention and Treatment of Cardiovascular and Cerebrovascular Diseases, Ministry of Education, Gannan Medical University, Ganzhou, China; ^2^Center for Evidence Based Medical and Clinical Research, First Affiliated Hospital of Gannan Medical University, Ganzhou, China; ^3^College of Pharmacy, Gannan Medical University, Ganzhou, China; ^4^Department of Biochemistry and Molecular Biology, Gannan Medical University, Ganzhou, China; ^5^Medical College, Jinggangshan University, Ji’an, China; ^6^Jiangxi Province Key Laboratory of Biomaterials and Biofabrication for Tissue Engineering, Gannan Medical University, Ganzhou, China

**Keywords:** Alzheimer’s disease, *Fibraurea recisa Pierre*, alkaloids, network pharmacology, AD pathology, heat-clearing and detoxifying traditional Chinese medicine

## Abstract

**Introduction:**

Heat-clearing and detoxifying Chinese medicines have been documented to have anti-Alzheimer’s disease (AD) activities according to the accumulated clinical experience and pharmacological research results in recent decades. In this study, *Fibraurea recisa Pierre* (FRP), the classic type of Heat-clearing and detoxifying Chinese medicine, was selected as the object of research.

**Methods:**

12 components with anti-AD activities were identified in FRP by a variety of methods, including silica gel column chromatography, multiple databases, and literature searches. Then, network pharmacology and molecular docking were adopted to systematically study the potential anti-AD mechanism of these compounds. Consequently, it was found that these 12 compounds could act on 235 anti-AD targets, of which AKT and other targets were the core targets. Meanwhile, among these 235 targets, 71 targets were identified to be significantly correlated with the pathology of amyloid beta (Aβ) and Tau.

**Results and discussion:**

In view of the analysis results of the network of active ingredients and targets, it was observed that palmatine, berberine, and other alkaloids in FRP were the key active ingredients for the treatment of AD. Further, Kyoto encyclopedia of genes and genomes (KEGG) pathway enrichment analysis revealed that the neuroactive ligand-receptor interaction pathway and PI3K-Akt signaling pathway were the most significant signaling pathways for FRP to play an anti-AD role. Findings in our study suggest that multiple primary active ingredients in FRP can play a multitarget anti-AD effect by regulating key physiological processes such as neurotransmitter transmission and anti-inflammation. Besides, key ingredients such as palmatine and berberine in FRP are expected to be excellent leading compounds of multitarget anti-AD drugs.

## Introduction

Alzheimer’s disease (AD) is a degenerative neurodegenerative disease that is progressive, complex, multifactorial, and incurable (Liu et al., 2013). According to statistics, people over the age of 65 are more likely to develop AD (Kim et al., 2020). These patients required careful treatment. Unfortunately, long-term treatment results are often disappointing, and healthcare providers and patients must bear high costs. According to the AD 2021 Facts and Figures report, a total of $355 billion is estimated to be spent in 2021 on care for older people with dementia in the US, and the COVID-19 pandemic has posed economic hardships (Alzheimer’s Association, 2021). AD patients with mild-to-moderate cognitive deficits remain most dependent on acetylcholinesterase inhibitors (AChEIs) as primary therapy (Lu et al., 2011; Zimmermann, 2013). These drugs are neurotransmitter regulators that can only temporarily relieve symptoms but cannot stop or reverse the progression of AD (Salomone et al., 2012). Long-term use of AChEIs has several shortcomings, including toxic side effects, patient intolerance, and high costs (Inglis, 2002). Therefore, there is a profound unmet need for AD therapy, and a novel strategy is urgently needed for the discovery of anti-AD drugs (Haake et al., 2020).

There has been much evidence indicating that multitarget drug therapy is more effective than one class of drugs for treating complex diseases such as malignancies (Cheng et al., 2019; Di et al., 2019). Developing highly active single-target anti-AD drugs based on classical targets has repeatedly failed to conquer AD. As a result, the construction of rationally formulated and efficacious anti-AD combinations or the development of multitargeted anti-AD drug molecules may be our new hope for conquering this complex disease (Yang W. T. et al., 2017; Oset-Gasque and Marco-Contelles, 2018). Although pharmacologists have made some impressive progress in these areas, there are still several pressing challenges to overcome (Rossi et al., 2021). For example, under drug safety, how to design anti-AD compounds to exert synergistic anti-AD effects and choose lead compounds more efficiently and quickly should be investigated.

To solve these problems, we focused on traditional Chinese medicine (TCM). Classical Chinese medicine has been proven to be safe and effective over many years of clinical use (Cordell and Colvard, 2012). According to modern pharmacological research, TCM contains not only a wide range of active ingredients but also a number of important small molecules with multiple functions. Consequently, TCM is expected to be a key entry point for developing a multitargeted therapeutic approach to AD (Islam et al., 2022). With years of clinical experience accrued, Professor Wang Yongyan proposed the “Kidney deficiency phlegm stasis –toxin brewing- collaterals disease” theory (Zhang and Wang, 2015). Based on this mechanism, using heat-clearing and detoxifying traditional Chinese medicine (HDTCM) to retard AD pathological progression is a wise choice (Shi et al., 2019). Since then, multiple research groups have confirmed that HDTCM can improve memorial and cognitive functions in AD mouse models and AD patients (Durairajan et al., 2017; Sun et al., 2018). Modern pharmacology shows that HDTCM contains a variety of potential anti-AD ingredients, and their mechanism of action involves multiple targets and pathways (Han et al., 2016; Park et al., 2017; Zhou et al., 2019; Wang Z. et al., 2020; Zhang et al., 2021a; Chen et al., 2022). In addition, several small molecules (such as forsythoside A, madecassic acid, and rhynchophylline) isolated from HDTCM sources were found to have anti-AD effects (Yan X. et al., 2017; Fu et al., 2021; Wong et al., 2021). Thus, HDTCM is a very promising and viable research direction for developing therapeutic regimens that target multiple aspects of AD. Hence, an in-depth study on the anti-AD properties of HDTCM is particularly effective for understanding the mechanism of AD pathogenesis, discovering AD therapeutic targets, and exploring novel candidates as potent drug candidates for AD.

*Fibraurea Recisa Pierre* (FRP) is a well-known HDTCM that is widely distributed in China, Vietnam, Laos, and Myanmar (Chen R. S. et al., 2015). According to the “Compendium of Materia Medica,” a famous Chinese pharmaceutical book written by Li Shizhen, “FRP is produced in the south of China, it looks like Fang Ji. Slang people often take this herb. Even if they eat poisonous food, they will not get sick.” In the present era, various pharmaceutical dosage forms of the crude alkaloid of FRP are available commercially in China, including tablets, injectables, and capsules. They have been widely used in the treatment of various diseases, such as gynecological inflammation, enteritis, and other diseases, and achieved successful results. The crude alkaloids of FRP have significant anti-AD effects on AD model rats, which may be due to some of the active compounds it contains (Xing et al., 2018; Zhang et al., 2020). For example, berberine and palmatine have been reported can exert various pharmacological activities, such as anti-inflammatory, chondroprotective, and neuroprotective properties (Cai et al., 2016; Singh et al., 2019; Jia et al., 2021; Ma et al., 2021). In addition, FRP contains other kinds of compounds, such as beta-sitosterol and oleanolic acid. These compounds were reported to play potential anti-AD roles *via* anti-inflammatory, and antioxidant effects (Song et al., 2016; Muhammad et al., 2017). Thus, FRP seems to be a promising agent for the treatment of AD. However, the possible mechanism involving FRP remains largely unknown and requires further elucidation.

**FIGURE 1:**
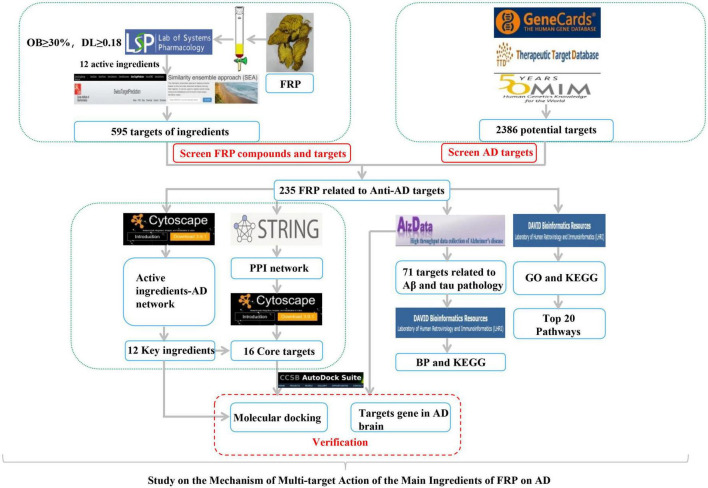
Flowchart of the study.

Network pharmacology has proven effective in explaining the mechanisms of TCM, which has complicated components and multiple targets (Hopkins, 2007; Li et al., 2007, 2011). Additionally, it could serve as the basis for the development of novel drugs (Lai et al., 2020). In this context, we first systematically analyze the key components and mechanisms of action of FRP, a classical HDTCM, by utilizing network pharmacology and molecular docking techniques. Based on the Alzdata database, there were 71 targets associated with tau and aβ pathologies. The research framework is shown in Figure 1. It is hoped that this study not only could offer guidance for the design of compounded anti-AD therapeutic regimens but also may provide a research basis for the development of novel multitarget anti-AD small molecules.

## Materials and methods

### Collection of main ingredients *Fibraurea recisa Pierre*

TCSMP (Ru et al., 2014): A relatively classical systemic pharmacology database and analysis platform for Chinese herbal medicines, collecting more than 500 Chinese herbal medicines with approximately 30,000 compounds, which is a more commonly used database for Chinese herbal medicine components. It has formed a more unified and systematic model by integrating pharmacodynamics, pharmacokinetics, and network and systemic analysis, thus providing scholars to study and analyze the interaction between Chinese herbal medicines and the organism at the systemic level and providing new directions for future target discovery, new drug development, and therapeutic strategies, and it is an important source for us to find data related to the active ingredients of FRP.

Uniprot (The UniProt Consortium, 2018) is the most extensive and informative protein database, which is composed of three sub-databases: Swiss Prot, TrEMBL, and PIR-PSD. The database currently includes a lot of protein and its function information from the literature. These data were mainly derived from the whole gene protein sequence obtained after genome sequencing of various species.

*Fibraurea recisa Pierre* (FRP) was purchased from Su Li Herbal Materials Company (Guangxi, China), which was authenticated by the Department of Pharmacy of Jinggangshan University by Professor Zhaochang Liang. Several major ingredients of FRP were separated and identified by using methods reported in the literature (Zhang et al., 2008; He et al., 2017). Other ingredients of FRP were retrieved by searching the relevant literature in PubMed,^1^ ZhiWang,^2^ WanFang,^3^ and WeiPu^4^ with “Huang Teng” and “*Fibraurea recisa Pierre*” as keywords. In addition, quality-control components of FRP in Chinese Pharmacopeia 2020 Edition were also considered. Then, all these ingredients were merged and analyzed to get the main ingredients of FRP, which may play critical roles in AD therapy most probably. Subsequently, these ingredients of FRP were retrieved and searched in TCMSP,^5^ and the relevant data of its main key components were supplemented. By combining with the common screening criteria for ingredient data in network pharmacology (the screening conditions were set as OB ≥ 30% and DL ≥ 0.18), the main ingredients and potential action targets of FRP could be obtained. Specifically, a few ingredients with low OB or DL were chosen as candidate ingredients because of their high bioactivity according to the literature. Meanwhile, the UniProt database^6^ was used to convert the gene name corresponding to each target name and organize the records.

### Prediction of the physicochemical properties of the main ingredients of the Chinese herbal medicine *Fibraurea recisa Pierre*

PubChem (Kim et al., 2016): A database containing the introduction of physical and chemical properties of a large number of compounds, these informations can be acquired by searching the molecule name, the molecular formula, and the molecular structure.

ADMETlab 2.0 (Su et al., 2007): A website for predicting the physicochemical properties of compounds, the ADME properties, and the toxicity correlations.

The common names of the main ingredients of FRP were retrieved from PubChem (see text footnote 1), and all their 2D structures were drawn by ChemDraw 15.0. Then, the SMILES strings of each main ingredients were obtained from PubChem (see text footnote 1) and imported into the ADMETlab 2.0^7^ to predict the relevant physicochemical properties.

### Screening the potential targets of the main active ingredients of *Fibraurea recisa Pierre*

SwissTargetPrediction (Daina et al., 2019): A database with updated data and new features for efficient prediction of protein targets of small molecules.

Search Server (SEA) (Keiser et al., 2007): A database that can be used to rapidly search large compound databases and to build cross-target similarity maps.

Swiss Target Prediction^8^ and SEA Search Server^9^ were employed to identify the ingredient-related target proteins according to the determined main active ingredients.

### Information collection and processing of disease targets

Online Mendelian Inheritance Database of Humans (OMIM) (Amberger and Hamosh, 2017): A comprehensive and authoritative human genetic database, which can provide information on approximately 15,000 genes, focusing on heritable or inherited genetic diseases and the molecular relationship between genetic variants and their dominant expression.

Drugbank (Wishart et al., 2008): A database for searching new drug targets, comparing drug structures, studying drug mechanisms, and exploring novel drugs.

TTD (Wang Y. et al., 2020): A database providing information on protein or nucleic acid targets of therapeutic value, including information on target-associated diseases, mediated biological pathways, etc.

PharmGKB (Pharmacogenetics and Pharmacogenomics Knowledge Base) (Whirl-Carrillo et al., 2021): A comprehensive resource that curates knowledge about the impact of genetic variation on drug response for clinicians and researchers.

Genecards database (Rebhan et al., 1998): A comprehensive medical database that provides rich biomedical data on genes and their products, including proteomic, genomic, and functional information on all known and predicted human genes, both genetic and functional.

Microbiology letter platform: An online platform for scientific data analysis and visualization.

STRING (Szklarczyk et al., 2017): A database for searching known protein-protein interactions (PPIs) and predicting PPIs, which allows people to predict potential PPIs.

Cytoscape (Shannon et al., 2003): A powerful software package, which can be used to visualize and analyzed biological networks, including pathways, PPIs, and protein-protein similarity networks. Besides, through third-party software extensions, the core functionality of Cytoscape could be expanded greatly.

CytoNCA (Tang et al., 2015): A plug-in Cytoscape, which is used for centralized analysis and evaluation of protein interaction network.

Molecular Complex Detection (MOCDE): A plug-in Cytoscape, which is used to identify sub-modules in the PPI network with biological significance.

By searching “Alzheimer’s disease” and “anti-Alzheimer’s disease” from OMIM,^10^ TTD,^11^ Drugbank,^12^ PharmGKB,^13^ and Genecards^14^ databases, the relevant targets can be obtained. Then, the final targets for the treatment of AD were collected after further processing and removing all duplicates targets. The targets corresponding to the main ingredients of Chinese herbal medicine, FRP, and the treatment of AD were used to obtain the intersection targets using the Microbiology letter platform.^15^ The intersection data were entered into the STRING database,^16^ the data were exported in TSV format, and the PPI relationship network was constructed using Cytoscape, thus revealing the visualized network structure map. Then, the core targets were screened using its plug-in CytoNCA, and the common anti-AD target gene clusters and pathway-related target genes of FRP were obtained using MOCDE.

### Construction of the drug-ingredient-target visualization network diagram

The collated data of the main ingredients and their potential targets of FRP were imported into Cytoscape (1 June 2022) to construct the drug-ingredient-target visualization network diagram. In the process of constructing the drug-ingredient-target visualization network diagram of FRP for AD, each target is constituted as a corresponding node, and in the layout options, a tool-analyzed Network is selected to obtain the degree of each data point. The larger the value of degree, the higher the participation of the node, and the stronger the credibility.

### Functional annotation of core targets and construction of pathway-target visualization maps

DAVID database (Huang da et al., 2009): A database now provides a comprehensive set of functional annotation tools for investigators to understand the biological meaning behind a large list of genes.

Gene Ontology (GO) is a classification system used to describe gene characteristics, gene product characteristics, and their main functions to describe three specific genes.

Biological process (BP) is used to describe the BP in which genes are involved, such as the regulation of growth factors.

Cellular component (CC) is used to describe the location of the gene product in the cell, e.g., in the mitochondria and nucleus.

Molecular function (MF) is used to describe the function of a single gene product and the function of multiple genes acting together.

Kyoto Encyclopedia of Genes and Genomes (KEGG) (Kanehisa and Goto, 2000): A database that is used to analyze the role of gene products in cellular metabolic pathways systematically and identify the metabolic pathways altered in the experiment.

The intersection target data were imported into the DAVID database,^17^ and GO and KEGG enrichment analyses (Chen J. et al., 2015) were performed on the intersection targets. Then the enrichment of BP, CC, MF, and KEGG pathway data was obtained. After filtering out the signaling pathway associated with AD in KEGG data, Cytoscape was then used to visualize the pathway-target map.

### Analysis of targets related to Alzheimer’s disease pathology

Alzdata (Xu et al., 2018) consisted of high-throughput omic data (e.g., Genomics, Transcriptomes, Proteomics, and Functional genomics), and high-confident functional data (e.g., neuroimaging screening, population-based longitudinal studies, and transgenic mouse phenotyping). The obtained human target proteins were input into the KEGG pathway database.

To evaluate the correlations between the target proteins of FRP against AD and AD pathology (Aβ and tau), their human gene symbols were entered and analyzed by using the AlzData database.^18^ The results were collected and entered into an Excel sheet. Subsequently, GO and KEGG pathway enrichment analyses were performed by using the DAVID tool. In addition, by using “AlzData’s” “Differential Expression” module, the normalized expression of targets of FRP against AD in control and AD groups of the GEO dataset were analyzed.

### Molecular docking

AutoDock (Morris and Lim-Wilby, 2008): A molecular simulation software, which was used to identify the molecular mechanism of interaction between the protein and the ligand most frequently.

RCSB PDB (Burley et al., 2021): A powerful new tool for exploring 3D structures of biological macromolecules for basic and applied research and education in fundamental biology, biomedicine, biotechnology, bioengineering, and energy sciences.

To reveal the binding modes and the strength between active ingredients and their protein targets, molecular docking simulation was performed using AutoDock 4.2. We collected the 2D structures of the main ingredients of FRP by PubChem,^19^ and then converted them to 3D and performed energy minimization. Next, the PDB formats of the core proteins corresponding to the core targets were downloaded from the RCSB PDB^20^ database. Next, water molecules and small molecule ligands were removed from the protein, hydrogen was added, and the active pockets in the 3D structure of the protein were identified by AutoDock Tools. Finally, the prepared protein models and ligand structures were used for molecular docking in the AutoDock Vina software (version 1.2.3, The Scripps Research Institute, San Diego, CA, USA). After performing the molecular docking simulation, the ligands were ranked by their docking scores, the higher the absolute value of the docking score indicates the stronger ability of the main ingredients to bind to the predicted important target proteins, and vice versa.

## Results and discussion

### Results of the collection of the main ingredients of *Fibraurea recisa Pierre* and disease targets

**FIGURE 2:**
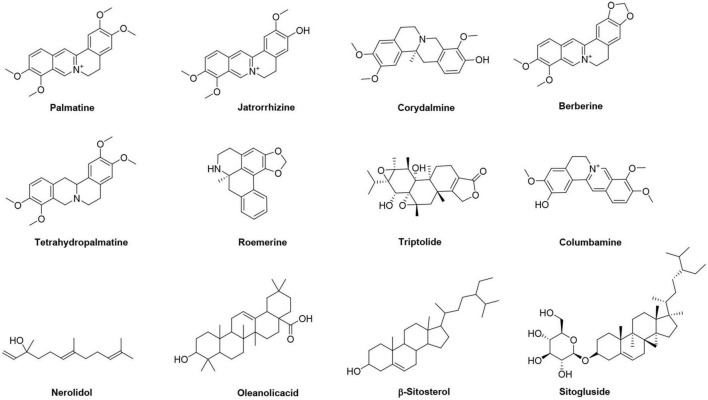
Structures of the main ingredients from *Fibraurea recisa Pierre*.

Five major chemical ingredients including berberine, palmatine, β-sitosterol, sitogluside, and jatrorrhizine were extracted from FRP by column chromatography and recrystallization. Their structures were confirmed *via* ^1^H NMR (Supplementary Figures 1–5), which is consistent with those reported in the literature (He et al., 2017; Wimmerová et al., 2017). Another 7 main ingredients of FRP were finally selected by using multiple online databases, including TCMSP, ZhiWang, WanFang, WeiPu, PubMed, and Chinese Pharmacopeia 2020 Edition. Their chemical structures were obtained from the PubChem database, as shown in Figure 2.

### The ADME properties of the main ingredients of *Fibraurea recisa Pierre*

The ADME-related properties of the main ingredients of FRP were evaluated in depth using the online tool ADMETlab 2.0, and all of them conformed to Lipinski’s five laws (*n* ≤ 1), including the topological acute surface area (TPSA) and solubility (LogS) indexes. These results indicated that the main ingredients of FRP possessed good permeability across the cell membrane, as shown in Supplementary Table 1.

### Screening of targets of the main ingredients of *Fibraurea recisa Pierre* in Alzheimer’s disease

**FIGURE 3:**
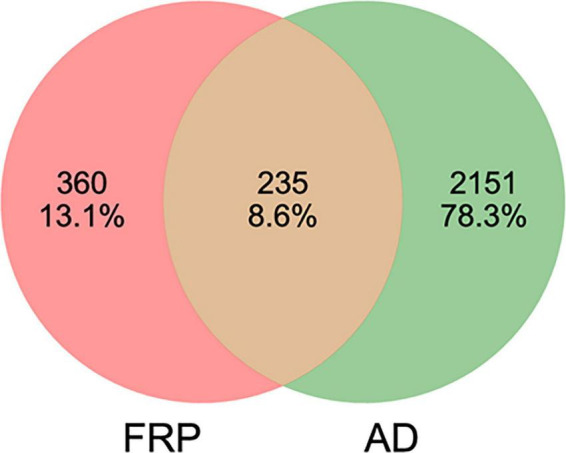
The intersection of FRP and AD targets.

A total of 595 relevant targets for FRP active ingredients were obtained from the SwissTargetPrediction database and SEA Search Server. Then, 2386 AD-related targets were obtained from OMIM, Drugbank, Genecards, TTD, and PharmGKB databases after performing duplicate removal. Subsequently, the potential targets of the active ingredient and AD-related disease targets were input into the online tool, and 235 potentially relevant targets for the treatment of AD were obtained for further research (Figure 3).

### Construction and analysis of the protein-protein interaction network

**FIGURE 4:**
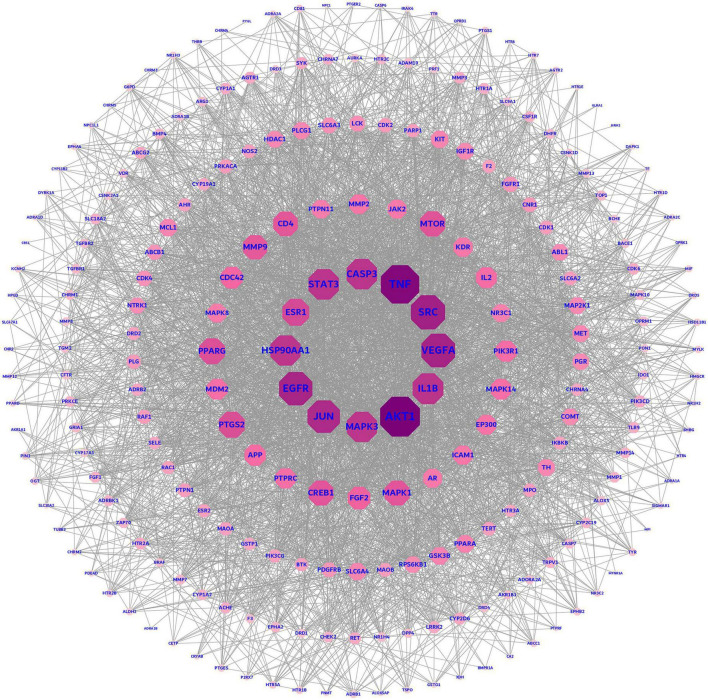
Protein-protein interaction (PPI) network diagram.

The intersection targets were entered into the String database. The organism was set to Homo sapiens, and the escaped nodes were hidden to finally obtain the data in TSV format, which was imported into Cytoscape 3.9.1 for processing. The result was presented below in Figure 4.

### Pathway-related target genes

**FIGURE 5:**
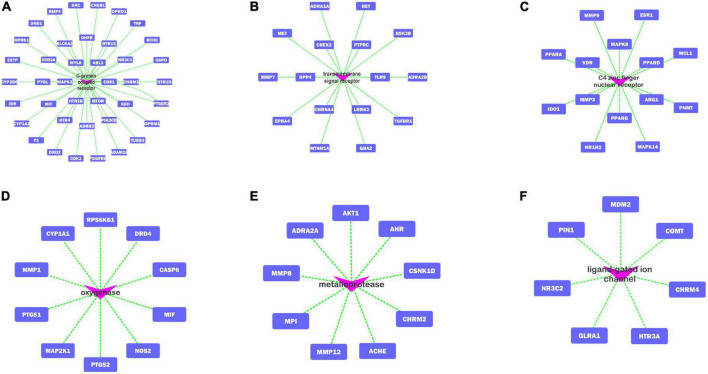
**(A–F)** Top 6 pathway-target gene clusters.

A total of 235 target proteins of FRP against AD were classified by Panther (Mi et al., 2007),^21^ and the results were summarized as follows. A total of 58 pathway-target gene clusters were obtained according to their cellular functions. The top 6 pathway-target gene clusters were the following (Figure 5): G-protein coupled receptors, transmembrane signal receptors, C4 zinc finger nuclear receptors, oxygenases, metalloprotease, and ligand-gated ion channels.

### Screening results of core targets

**FIGURE 6:**
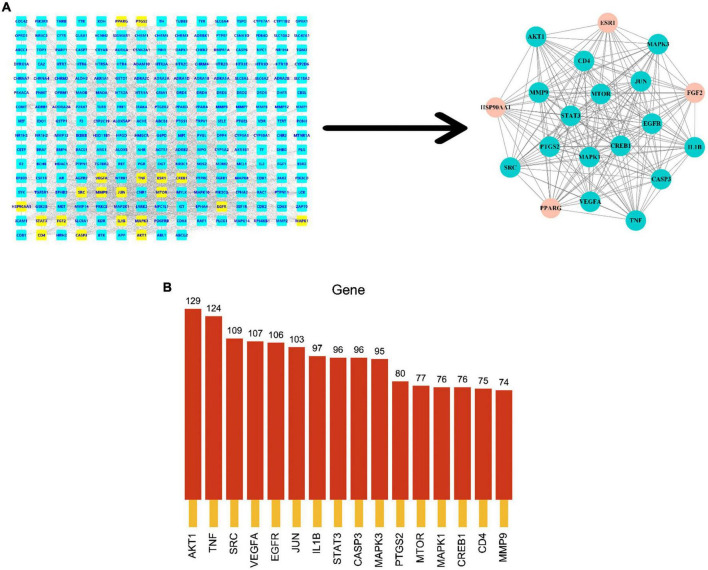
Screening of core targets. **(A)** Shows the process of secondary median screening, and the dark green part indicates the core target genes obtained from the screening. The core targets after the secondary screening were arranged in the order of the degree values from the largest to smallest, and the bar graph was drawn to obtain **(B)**.

To explore the relationship between potential predicted targets of the major components of FRP and AD disease targets, two screens were performed in Cytoscape using the CytoNCA plugin to obtain the final core targets with a median of twofold, as shown in Figure 6. The FRP in the figure shows the final targets after the two screens. The key targets screened in Figure 6A were ranked according to the degree values and histograms were plotted, as shown in Figure 6B. Based on these results, AKT1, TNF, STAT3, JUN, and EGFR were closely related to other targets in the PPI network, and presumably, they were identified as the most likely to be important potential targets against AD.

### Common target clusters of main anti-Alzheimer’s disease ingredients

**FIGURE 7:**
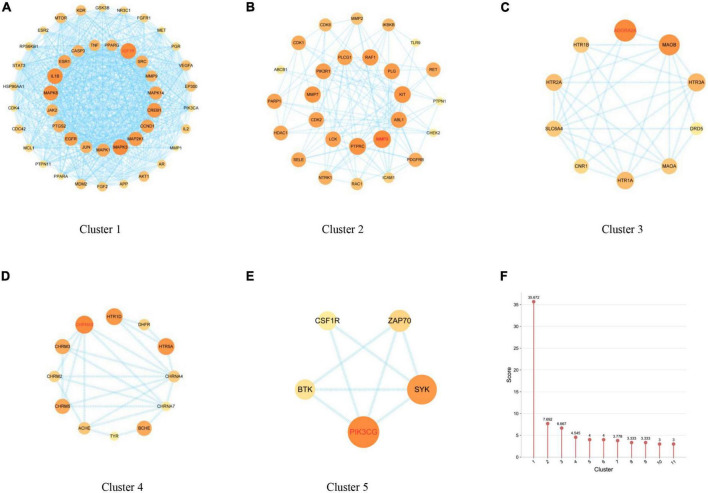
**(A–E)** Discovery of clusters 1–5 using MCODE, which identifies densely connected regions. The seed nodes of each cluster are indicated in red font. **(F)** Comparison of MCODE scores for different clusters.

Network analysis of the anti-AD PPI network of the main ingredients of FRP by MCODE (k-core = 2) showed that these clusters may be relevant to AD treatment. As shown in Figure 7, cluster 1 contains 42 nodes and 86 edges with a score of 33.463, seeded by AKT1, which have been confirmed to play a vital role in anti-AD pathobiology, including regulation of intracellular reduction-oxidation balance, activation of autophagy, anti-inflammatory actions and neuroprotection, etc. (Figure 7A); cluster 2 contains 18 nodes and 89 edges with a score of 10.471, seeded by SLC6A3, which is associated with learning and memory (Figure 7B); cluster 3 contains 30 nodes and 126 edges with a score of 8.690, seeded by MMP1, which is associated with anti-inflammation (Figure 7C); cluster 4 contains 6 nodes and 13 edges with a score of 5.200, seeded by PIK3CG, which is associated with atheromatosis (Figure 7D); cluster 5 contains 4 nodes and 6 edges with a score of 4.000 points (Figure 7E); cluster 6 contains 13 nodes and 21 edges with a score of 3.500, seeded by AKT1; and cluster 7 contains 4 nodes and 5 edges with a score of 3.330, seeded by AKT1. Cluster 8 contains 3 nodes and 3 edges with a score of 3.000; Cluster 9 contains 9 nodes and 12 edges with a score of 3.000, seeded by AKT1. Cluster 10 contains 3 nodes and 3 edges with a score of 3.000, seeded by AKT1. Cluster 11 contains 7 nodes and 9 edges with a score of 3.000, seeded by the node AKT1. A list of the respective MCODE scores is shown in Figure 7F.

### Construction of *Fibraurea recisa Pierre*- main ingredients-target visualization map

**FIGURE 8:**
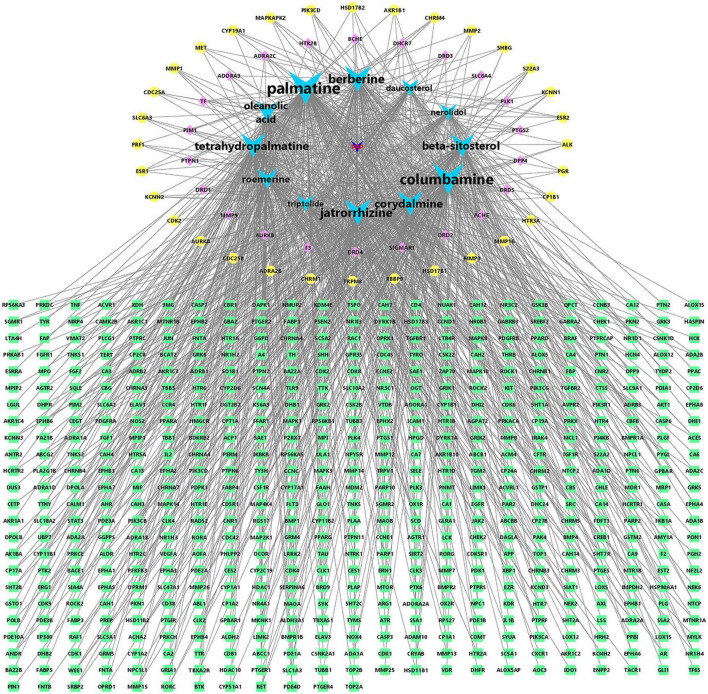
*Fibraurea recisa Pierre* (FRP)-active ingredient-target visualization. Green is the gene with a degree of 3, yellow is the gene with a degree of 4, pink is the gene with a degree of 5–8, blue is the main ingredients with a degree of 46–156, and dark blue is the traditional Chinese medicine FRP.

To explore the mechanism of action of FRP for the treatment of AD, 235 drug-disease intersection gene targets and 12 main ingredients in FRP were used to construct the major ingredient target AD target network. As shown in Figure 8, all of these compounds were associated with multiple targets, and 1,086 component-target associations existed between the 12 compounds and 235 targets. The average number of targets per compound was 18.58, and the average composition degree per target was 1.82. These results clearly support that the anti-AD mechanism of FRP has multi-components and multi-targets characteristics. As shown in Figure 8, Palmatine (degree = 156) has the highest number of targets, followed by columbamine (degree = 143), jatrorrhizine (degree = 109), berberine (degree = 109), corydalmine (degree = 100), tetrahydropalmatine (degree = 99), β-sitosterol (degree = 71) and oleanolic acid (degree = 77). The above results suggest that alkaloids from FRP are most likely to be the key components of AD therapy, besides β-sitosterol and oleanolic acid may also have multitarget anti-AD potential.

### Gene ontology and Kyoto encyclopedia of genes and genomes enrichment analysis

**FIGURE 9:**
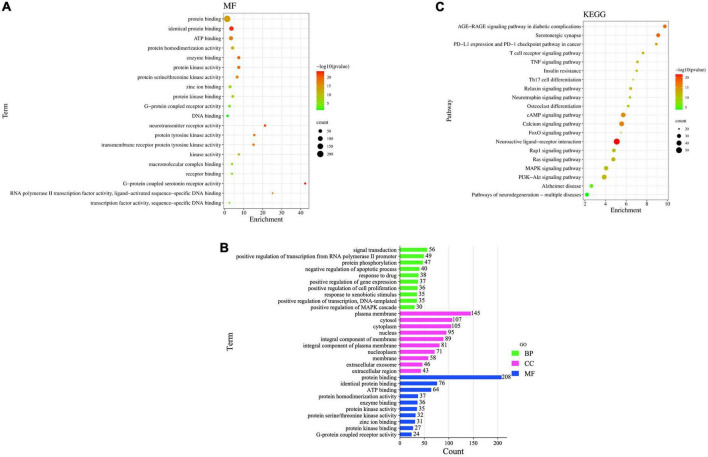
**(A)** MF enrichment bubble diagram, **(B)** GO enrichment bar graph, **(C)** KEGG pathway enrichment analysis.

Gene Ontology enrichment analysis and KEGG pathway enrichment analysis were performed using David on the intersecting targets, and 235 MFs entries were screened out, the 20 closely related MFs are shown in Figure 9A, Including (GO:0005515) protein binding, (GO:0042802) identical protein binding, (GO:0005524) ATP binding, (GO:0042803) protein homodimerization activity, (GO:0019899) enzyme binding, (GO:0004672) protein kinase activity, (GO:0004674) protein serine/threonine kinase activity, (GO:0008270) zinc ion binding, (GO:0019901) protein kinase binding al. These results further confirm that the main ingredients in FRP can exert anti-AD effects by acting on multiple targets and biological functions.

Gene Ontology enrichment analysis got 1,264 entries, of which 947 were BP entries, 111 were CC entries, and 206 were MF entries. As shown in Figure 9B, the common targets of main ingredients and AD in FRP are mainly enriched in signal transduction, positive regulation of transcription from RNA polymerase II promoter, protein phosphorylation, negative regulation of the apoptotic process, response to the drug, positive regulation of gene expression, positive regulation of cell proliferation, response to xenobiotic stimulus, positive regulation of transcription, DNA-templated, positive regulation of MAPK cascade. For example, the signal transduction pathway involves 56 target genes, of which 8 are related to target genes, namely 1L1B, AKT1, MAPK1, CREB1, EGFR, CD4, STAT3, and SRC MFs are mainly enriched in protein binding, identical protein binding, ATP binding, protein homodimerization activity, enzyme binding, protein kinase activity, protein serine/threonine kinase activity, zinc ion binding, protein kinase binding, G-protein coupled receptor activity, etc.; Cellular composition is mainly enriched in the plasma membrane, cytosol, cytoplasm, nucleus, an integral component of membrane, integral component of the plasma membrane, nucleoplasm, membrane, extracellular exosome, extracellular region, etc.

To further evaluate the functions of these overlapping target genes, 235 drug-disease intersection gene targets were filtered through the KEGG pathway. Pathway enrichment analysis revealed that these genes were enriched in 170 signaling pathways, and the top 20 signaling pathways most closely related to AD were the following (Supplementary Table 2): AD-related pathways include neuroactive ligand-receptor interaction, PI3K-Akt signaling pathway, calcium signaling pathway, cAMP signaling pathway, MAPK signaling pathway, Ras signaling pathway, serotonergic synapse, pathways of neurodegeneration multiple diseases, Rap1 signaling pathway, AD and AGE-RAGE signaling pathway in diabetic complications. To identify biological target-related signaling pathways associated with anti-AD, we used DAVID to identify enriched signaling pathways of 235 drug-disease intersection gene targets. The important pathways involved in the KEGG pathway were neuroactive ligand-receptor interaction (hsa04080), PI3K-Akt signaling pathway (hsa04151), calcium signaling pathway (hsa04020), cAMP signaling pathway (hsa04024), MAPK signaling pathway (hsa04010), Ras signaling pathway (hsa04014), serotonergic synapse (hsa04726), and pathways of neurodegeneration–multiple diseases (hsa05022), Rap1 signaling pathway (hsa04015) and Alzheimer’s disease (hsa05010). The KEGG pathway enrichment analysis is listed in Supplementary Table 2. The most important enriched pathways were the neuroactive ligand-receptor interaction pathway (hsa04080, *P*-value = 1.78E-22) and the PI3K-Akt signaling pathway (hsa04151, *P*-value = 7.33816E-15), which are related to human diseases and are mainly associated with Figure 9C.

### “Pathway-vital target” network model and analysis

**FIGURE 10:**
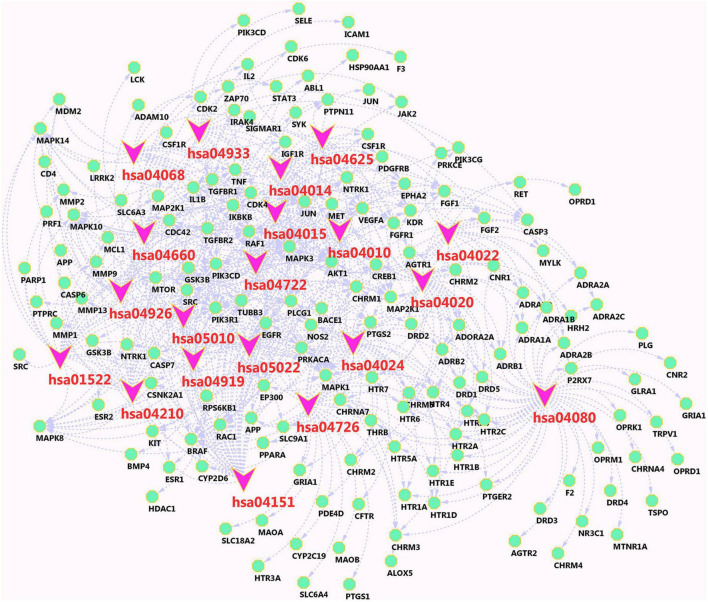
Passage-target visualization diagram. Pink nodes are pathways, and green nodes are related target genes.

The network of main components and their related targets of Chinese herbal medicine FRP is shown in Figure 10, containing 182 nodes and the top 20 KEGG pathways associated with 235 targets and 553 edges. In particular, the PI3K-Akt signaling pathway (hsa04151) shows a higher number of connections (degree = 38), which includes the target genes VEGFA, MAPK3, AKT1, MAPK1, CREB1, EGFR, ADORA2A, MTOR, and PIK3CG, et al. Neuroactive ligand-receptor interaction (hsa04080) has the highest number of connections (degree = 51), which includes the target genes, namely, ADORA2A, THRB, CHRM1, CHRM4, CHRM5, HTR2B, HTR2C, ADRA1D, HTR2A, ADRA1B, HTR4, and NR3C1, et al.

### Bioinformatics analysis of alkaloid targets associated with amyloid beta and tau pathology

**FIGURE 11:**
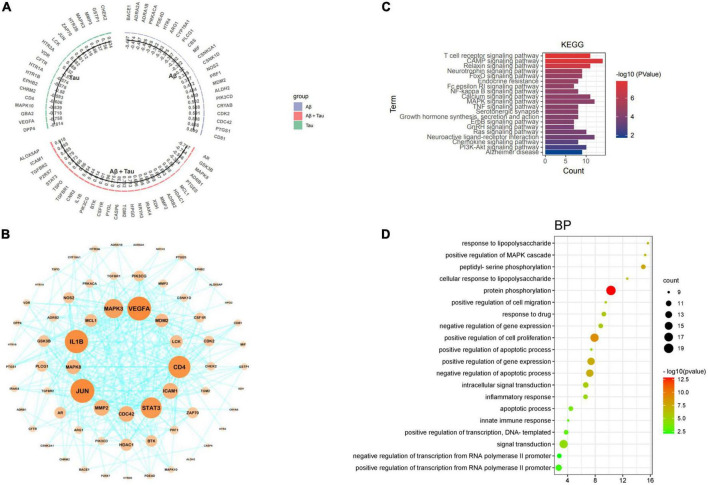
**(A)** Bar graph showing target genes with tau and Aβ information. **(B)** Bioinformatics analysis of FRP targets associated with tau and Aβ pathology. **(C)** Tau and Aβ-related targets of the KEGG pathway. **(D)** Enrichment analysis identified bubble maps associated with the top 20 biological processes (BP).

To further validate the relationship between the potential targets of the Chinese herbal medicine FRP and the potential mechanisms of AD (Aβ and Tau), the intersecting genes were entered into the AlzDate database. Among these targets, 71 were associated with Aβ, Tau, Aβ, and Tau (Figure 11A), specifically, PIK3CD, CDC42, PDE4D, HTR4, ADRA1B, ARG1, PTGS1, PRF1, ADRA2A, CD81, CRYAB, BACE1, CDK2, MDM2, ALDH2, CYP19A1, NOS2, CSNK1D, PRKACA, CSNK2A1, PLCG1, CBS, and MIF were significantly associated with Aβ pathology, EPHB2, LCK, JUN, ZAP70, DPP4, HTR2B, MAPK10, HTR1A, VEGFA, HTR1B, CFTR, CHRM2, GBA2, GSTP1, MMP3, HTR3A, CD4, VDR, MAPK3, and CHEK2 were significantly associated with Tau pathology. Significantly, CNR2, HDAC1, MCL1, XDH, IL1B, TGFBR2, GSK3B, CASP6, HPGD, ADRB2, CSF1R, PIK3CG, TGFBR1, PTGES, MAPK8, ADRB1, NR1H3, IRAK4, P2RX7, ALOX5AP, PYGL, MMP2, STAT3, ICAM1, TGM2, TSPO, AR, and BTK equals Aβ and Tau pathology. At the same time, 71 target genes were imported into the STRING database for processing, and the processed data were imported into Cytoscape, which was arranged according to the size of nodes. A total of 67 nodes and 334 edges were found. JUN, VEGFA, IL1B, CD4, STAT3, MAPK3, MMP2, ICAM1, CDC42, MAPK8, MDM2, LCK, MCL1, and HDAC1 were identified as the core targets sorted by degree (Figure 11B), which also confirmed the multitargeting of FRP against AD. According to KEGG pathway analysis, the cAMP signaling pathway (hsa04024), MAPK signaling pathway (hsa04010), Neuroactive ligand-receptor interaction (hsa04080), T-cell receptor signaling pathway (hsa04660), Relaxin signaling pathway (hsa04926), Calcium signaling pathway (hsa04020), Ras signaling pathway (hsa04014), PI3K-Akt signaling pathway (hsa04151), Neurotrophin signaling pathway (hsa04722) are highly enriched (Figure 11C). MAPK10, BACE1, GSK3B, MAPK8, CSNK2A1, NOS2, IL1B, PIK3CD, and MAPK3 were enriched in the AD pathway (hsa05010). David further analyzed 71 target genes, the prediction of biological processes (BP), including protein phosphorylation (GO:0006468), positive regulation of cell proliferation (GO:0008284), peptidyl-serine phosphorylation (GO:0018105), Positive regulation of gene expression (GO:0010628), negative regulation of apoptotic process (GO:0043066), response to lipopolysaccharide (GO:0032496), positive regulation of MAPK cascade (GO:0043410), positive regulation of MAP kinase activity (GO:0043406) (Figure 11D). In particular, protein phosphorylation (GO:0006468) shows the highest target connectivity, including the core target genes MAPK3 and PIK3CG.

### Molecular docking

**FIGURE 12:**
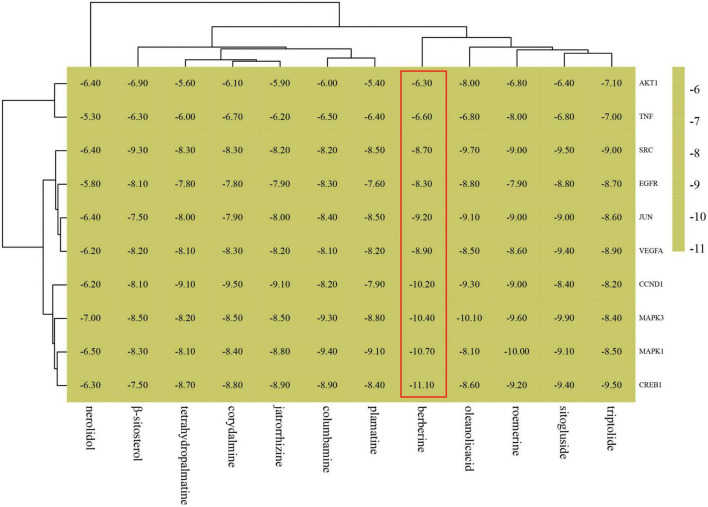
Binding energy thermogram of active ingredients of FRP with target proteins.

**FIGURE 13:**
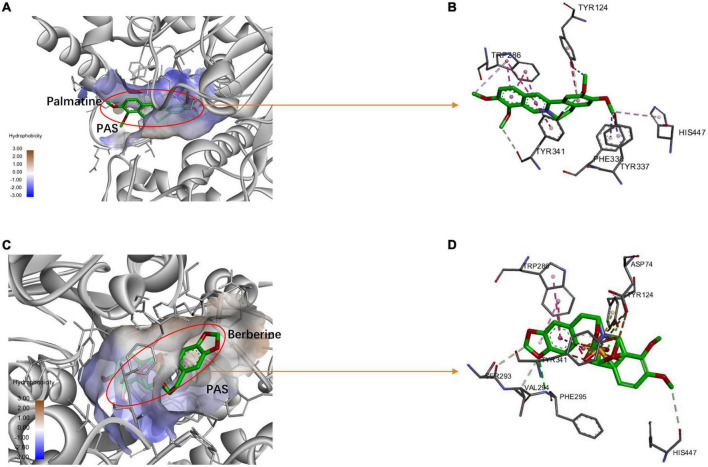
The protein-ligand of the docking simulation: **(A)** Palmatine and 4EY7 (3D); **(B)** Palmatine and 4EY7 (2D); **(C)** Berberine and 4EY7 (3D); **(D)** Berberine and 4EY7 (2D).

Ten targets (AKT1, TNF, SRC, EGFR, JUN, VEGFA, CND1, MAPK3, MAPK1, and CREB1) were chosen according to the results of the PPI network as the core targets of FRP for the treatment of AD. Therefore, molecular docking was performed by AutoDock software to simulate the interaction between the main ingredients of FRP with these 10 core anti-AD target proteins. The coordinates and box size for molecular docking were depicted in Supplementary Table 3. Then, the docking results were made into a heatmap as shown below (Figure 12). Of those compounds, berberine exhibits the highest binding energy to the binding pocket of core target proteins. Meanwhile, other alkaloids, such as columbamine, roemerine, and palmatine show a slightly weaker binding affinity with core target proteins. In addition, oleanolic acid and β-sitogluside also have good affinity to the target proteins. Furthermore, the molecular simulation was used to verify the binding ability of the alkaloid compounds to the classical therapeutic target (AChE) and explore their accurate binding modes. As shown in Figure 13, palmatine was docked into the PAS site of AChE (PDB ID = 4EY7) through hydrogen bonding, van der Waals interaction, PI donor hydrogen bonding, Pi-Pi Stacked, and Pi-Pi T-shaped, based on the molecular docking studies, more rational design and structural modification can be performed on the palmatine in the future research, thus enhancing its anti-AD effect. And berberine was docked into the PAS site of AChE (PDB ID = 4EY7) through Pi-Cation, Pi-Sulfer, and Pi-Pi Stacked.

## Discussion

According to the theory of Chinese medicine, AD is considered a multitargeted systemic disease, which is consistent with the contemporary view of modern Western medicine coincidentally (Rollo et al., 2016). Several clinical trials have shown that HDTCM can improve cognition in AD patients. FRP belongs to a kind of classical HDTCM with obvious anti-inflammatory activity and antioxidant activity. In this study, by utilizing network pharmacology and molecular docking, we revealed the potential therapeutic targets of FRP for treating AD. Meanwhile, the key active components of FRP for anti-AD activity were identified.

Although network pharmacology has become a useful tool to lend insight into the mechanisms of TCM. Even so, there are still some problems to overcome. Based on the newly bibliometric analysis results of network pharmacology in TCM, determining the key ingredients in TCM (Miao et al., 2022) just based solely on data from limited databases is not sufficient, which may impact the quality of follow-up research. Researchers believe that this phenomenon occurred because the public databases do not reflect the difference in the composition and content of TCM compounds. Meanwhile, it is necessary to focus on basis of TCM’s properties, and In-depth research on the key components’ pharmacological activity is urgently needed. According to the previous literature, we isolated and identified 5 ingredients of FRP by silica gel TLC and recrystallization (Zhang et al., 2008). Additionally, the other 7 ingredients were chosen after a systematic search from multiple aspects. Finally, a total of 12 ingredients from FRP were selected for further investigation. From the compound-target-disease target network, 235 potential targets were identified. Of these, 16 potential targets were chosen as core targets based on the degree values in the PPI network. These core targets might play critical roles in the anti-AD processes by FRP, and they are also expected to become novel therapeutic targets for multitargeted treatment most likely. Meanwhile, 6 kinds of alkaloids (palmatine, columbamine, jatrorrhizine, tetrahydropalmatine, and romaine) were speculated to play significant roles in the prevention of AD progression. Based on the ADMET lab 2.0’s prediction results, all these alkaloids meet Lipinski’s rule of five with no violations. In addition, Total Polar Surface Area (TPSA) Studies have shown that all these alkaloids characterize significant permeability in the cellular plasma membrane.

Based on literature reports, nitrogen-containing heterocyclic substrates often have high therapeutic potential to treat neurodegenerative diseases, especially AD. In this study, palmatine (degree = 156) showed the highest number of anti-AD targets, but the current study on its anti-AD properties is limited. Per the existing literature, palmatine could attenuate LPS-induced inflammation by inhibiting ERK1/2, P38, and Akt/NF-kB signaling pathways (Ma et al., 2021). Besides, by decreasing its anti-inflammatory and antioxidative effects, palmatine could exert its neuroprotective action in the rat model of brain I/R injury (Tang et al., 2021). In addition, autophagy could also be promoted by palmatine through activation of the AMPK/mTOR signaling pathway that may contribute to its antioxidant and neuroprotective activity (Lin et al., 2022). Additionally, previous studies have shown that palmatine could possess significant AChE inhibitory activity *in vitro* (Chaves et al., 2020; Song et al., 2021). These findings suggested that palmatine is highly possible to be a promising leading compound for AD treatment. Moreover, palmatine was the most abundant alkaloid of FRP, which means we don’t have to worry about palmatine being insufficient for widely used and in-depth development.

The existing literature shows that berberine has attracted much research, due to its multiple pharmacology activities and favorable safety profile (Ai et al., 2021; Fang et al., 2022). In recent years, more targets for the anti-AD effect of berberine have been continuously discovered (Wong et al., 2020; Ye et al., 2021). Meanwhile, only a small number of studies have been documented on the anti-AD effect of columbamine, and corydalmine (Ingkaninan et al., 2006; Li et al., 2019). Thus, using these alkaloids for AD treatment could be explored deeply in future research. Besides, several research groups have evaluated the effects of palmatine, berberine, and their combination on AChE. As a result, the combination of palmatine and berberine exerted stronger activity on AChE activity *in vitro* enzymatic activity assay (Balkrishna et al., 2019). In addition, we have carried out further studies on the main ingredients of FRP. Even though these ingredients have been shown potential anti-AD biological activities. However, many of them still have some shortcomings to overcome (e.g., poor bioavailability, limited CNS penetration) (Cui et al., 2015; Fan et al., 2019). Several studies have reported that modifying molecular structures rationally or using nanotechnology to improve the drug delivery system may increase their water solubility and bioavailability (Xu et al., 2020).

Therefore, to design innovative anti-AD drugs more efficiently and reasonably, it is essential to clarify the target and molecular mechanism of these main ingredients of FRP (Baig et al., 2016; Aarthy et al., 2017). Besides, this work was also beneficial to the design of newer anti-AD combination therapies. Therefore, it is necessary to reveal the core anti-AD targets and critical signaling pathways by FRP. According to the results of our study, FRP can treat AD by regulating multitarget with multi-components. The PPI Network of the active compound targets against AD was analyzed by MCODE to obtain 7 clusters. These clusters might play important roles in the anti-AD effect of FRP. Moreover, to investigate FRP’s potential mechanism of action on anti-AD, the DAVID database was utilized to perform enrichment GO and KEGG pathway enrichment analysis. The results showed that Neuroactive ligand-receptor interaction (degree = 51), PI3K-Akt signaling pathway (degree = 38), Calcium signaling pathway (degree = 37), and cAMP signaling pathway (degree = 35) were the top 4 significantly enriched pathways. Therein, Neuroactive ligand-receptor interaction (hsa04151), Calcium signaling pathway (hsa04020), and cAMP signaling pathway (hsa04024) are frequently associated with neurogenesis transmission. Several targets (ADORA2A, HTR4, DRD1, et al.) are involved in all these 4 pathways. According to recent studies, elevated expression of ADORA2A was found in the brain tissue of AD mouse models and AD patients (Ferreira et al., 2015). After treatment with the selective A2AR antagonist in the triple transgenic mouse model, the level of VGluT1 expression could be upregulated, and the number of synaptic A2AR in the hippocampus (HP) increase was observed, meanwhile, the cognition of the mouse was improved (Temido-Ferreira et al., 2020). These results indicated that ADORA2A might be a promising therapeutic target for AD (Silva et al., 2018). Besides, the PI3K-Akt signaling pathway (degree = 41) has the most associations with targets. Previous studies have suggested that this signaling pathway is one of the most vital signaling pathways associated with inflammation, immunity, and apoptosis in the human body (Sun et al., 2021; Merighi et al., 2022). The results of our study indicate that the major components of FRP can take effect on multiple targets of this signaling pathway (e.g., AKT1, EGFR, IGF1R, et al.). Among these predicted targets, 4 targets were classified as core targets including AKT1 (degree = 16), EGFR (degree = 8), VEGFA (degree = 7), and CREB1 (degree = 4). According to their degree values in the PPI network. Akt is an attractive therapeutic target for AD. Previous studies have shown that the PI3K activation could activate AKT by phosphorylating it at Thr308 and Ser473, following that, activated AKT phosphorylated GSK-3β (Hu et al., 2020), which inhibits glycogen synthase kinase-3β (GSK-3β) (Ma et al., 2008; Weako et al., 2021). Based on the *in vivo* experiment in the ICV-STZ rat model of AD, modulation of the PI3K/Akt/GSK3β signaling pathway could diminish oxidative stress, neuro-inflammation, and apoptosis, Moreover, amyloid plaque number and phosphorylated tau expression were marked decrement (Nishizaki, 2018). Besides, it has been reported that the intra-hippocampal administration of Aβ_1–42_ to adult rats could disrupt the reduction-oxidation (redox) balance and significantly affect neurotransmitter dysfunction, synaptic dysfunction, and cognitive dysfunction (Yamini et al., 2022). When rapamycin was administered to the Aβ_1–42_-treated rats, Akt1 could be activated, which further increased the expression levels of synaptic markers and neurotransmitter markers. Meanwhile, autophagy could be activated by rapamycin *via* PI3K/mTOR signaling pathway, and thus, protects hippocampal neurons from degeneration by decreasing the levels of prooxidants (Kotagale et al., 2020). These results not only indicated that autophagy might be sufficient to reverse the redox imbalance induced by Aβ_1–42_, but also reveal the crucial role of Akt1 and PI3K in neuroprotection. In addition, we have noticed a very recent bioinformatics analysis by Singh et al. (2017). By using the publicly available GEO database, they analyzed the miRNA/mRNA expression profiles of AD patients and controls in the Asian population (ID: GSE131617, GSE36980, GSE139384, and GSE120584). Go enrichment analysis and KEGG pathway analysis were performed on differentially expressed genes in the four brain regions. Results revealed that these genes in the Frontal Cortex (FC), Temporal Cortex (TC), and HP were enriched in the biological pathophysiology relevant to neurotransmitters with receptors. Furthermore, the maximum number of core differential genes were observed in HP areas, and these genes were mostly enriched in the neuroactive ligand-receptor interaction pathway. Interestingly, this pathway has been identified as an important signaling pathway in the treatment of AD by FRP as previously shown. Besides, the genes in EC were enriched in immunity, inflammation, apoptosis, and other signaling pathways. As the antecedent show, FRP can exert anti-inflammatory through multiple signaling pathways. To a certain extent, these results demonstrate that FRP has a high potential for the treatment of AD. As is well-known, phosphorylated tau tangles and amyloid-β plaques are the key pathological feature of AD. In further studies, we examined the relationship between FRP targets and these two pathologies (Aβ and tau). Up to 71 out of 235 overlapping targets showed significant correlations with Aβ, tau, or Aβ and tau. These targets were enriched in several signaling pathways, such as the cAMP signaling pathway, MAPK signaling pathway, and neuroactive ligand-receptor interaction pathway. Coincidentally, according to the existing research, these three BP may play important roles in the pathogenesis and maintenance of AD.

According to the discussions above, our network pharmacology analysis predicted that the main ingredients of FRP have multiple potential anti-AD properties, including neuroprotective, anti-inflammatory, and anti-neuroinflammation properties that may make it useful for establishing multitargets anti-AD therapy. To further verify the results of network pharmacology, the interactions between the key ingredients and core targets were analyzed by molecular docking. The docking score results demonstrated that all these compounds have moderate to strong binding affinity for these potential targets. Among these 12 compounds, the highest docking score was achieved by berberine. Other protoberberine alkaloids in FRP, such as palmatine, jatrorrhizine, and columbamine could also show a moderate binding affinity with core target proteins. Existing research suggests that all these alkaloids could possess various biological activities, including anti-oxidants, anti-inflammatory, and neuroprotective, which may be beneficial for the treatment of AD (Wang et al., 2017; Yan X. et al., 2017; Li et al., 2019; Long et al., 2019; Zhong et al., 2021). Besides, oleanolic acid exhibited high binding to most of the core targets. Although this compound was found to possess a variety of biological activities, such as anti-inflammatory, antioxidant, and neuroprotection (Zhang et al., 2021b; Zeruo et al., 2022). Due to its poor pharmacokinetic parameters, its clinical application has been limited (Gudoityte et al., 2021). Another kind of steroidal compound, sitogluside, also showed good docking scores. However, very few previous studies have examined its anti-AD properties. Therefore, it is perhaps deserved to be investigated in future research. In recent years, molecular docking technology has become a powerful tool for rational drug design. According to the descending order of degree values of the key ingredients in the “ingredients-targets” network and their molecular docking scores. The top-ranked active ingredients (palmatine and berberine) have been selected out as the most potent anti-AD lead compound. Analysis results of their molecular structures indicated that the modification of these two alkaloids could be exerted at multiple sites, which may probably improve their bioavailability and biological activity (Shan et al., 2011; Yan B. et al., 2017; Liang et al., 2021; Castellano et al., 2022). Notably, the rational structural modifications of AChE inhibitors were still the primary route to the discovery of novel One-molecule-multi-target anti-AD drugs at this stage (Rossi et al., 2021; Sang et al., 2022; Turgutalp et al., 2022). Since these two alkaloids’ high potential *in vitro* acetylcholinesterase inhibitory activity had been verified by other researchers (Jia et al., 2021; Tang et al., 2021; Miao et al., 2022). Molecular docking analysis was used to predict the binding mode between these two compounds and AChE. The docking results were not only further confirming their AChE inhibition mechanism but also provided a basis for the rational design of multi-target anti-AD drugs.

In the further studies, we noted that many other herbal medicines (*Coptis chinensis Franch*, *Phellodendron amurense*, and *Berberis sargentiana Schneid*, et al.) also reportedly contain some of the same alkaloids compared with FRP (Wang et al., 2009; Yang Y. et al., 2017; Meng et al., 2018; Adefegha et al., 2021). According to our network pharmacology analysis, palmatine was identified as the most effective anti-AD active component among these alkaloids. Since the palmatine content of these herbal medicines are far less than that of FRP, it is reasonable to believe that FRP could exert more potent anti-AD activity than any of them.

## Conclusion

In this study, we successfully investigated the key active components and molecular mechanisms of FRP implicated in the treatment of AD by network pharmacology analyses and molecular docking. This research identified 12 ingredients of FRP treatment of AD by targeting 235 targets *via* multiple pathways, which were concentrated on the neuroactive ligand-receptor interaction, PI3K-Akt, calcium signaling, and cAMP signaling pathways. Meanwhile, 16 core targets were identified by PPI network analysis. According to the extraction experiment and molecular docking results, alkaloids of FRP, such as palmatine and berberine, were not only the most abundant components in FRP but also had the best anti-AD activity. Moreover, these components showed promising enzyme activity against AChE, which currently remains the foremost therapeutic target for AD. In conclusion, the results of this study not only further verified that FRP in AD treatment has the characteristics of “multiple components, multiple targets, and multiple pathways” through network pharmacology but also identified possible leading compounds with potential multi anti-AD activity. Moreover, our research opens the door for the potential application of HDTCM to multitargeted AD therapy. Additionally, future *in vivo* and *in vitro* experiments are needed to be performed to verify the results of the present study.

## Data availability statement

The original contributions presented in this study are included in the article/Supplementary material, further inquiries can be directed to the corresponding authors.

## Author contributions

YW conceived and designed the review. SsW and YxM wrote the manuscript. YsH, YhH, and YpH revised the manuscript. SsW, YxM, YpH, YsH, YhH, and YW approved the final version of the manuscript. All authors contributed to the article and approved the submitted version.

## Footnotes

1 https://www.ncbi.nlm.nih.gov (accessed May 16, 2022).

2 https://cnki.net (accessed May 14, 2022).

3 https://www.wanfangdata.com.cn/ (accessed May 15, 2022).

4 http://www.cqvip.com/ (accessed May 16, 2022).

5 https://old.tcmsp-e.com/ (accessed May 16, 2022).

6 https://www.uniprot.org/ (accessed May 16, 2022).

7 https://admetmesh.scbdd.com/ (accessed May 16, 2022).

8 http://www.swisstargetprediction.ch/index.php (accessed May 17, 2022).

9 https://sea.bkslab.org/ (accessed May 17, 2022).

10 https://omim.org/ (accessed May 18, 2022).

11 http://db.idrblab.net/ (accessed May 18, 2022).

12 https://go.drugbank.com/ (accessed May 19, 2022).

13 https://www.pharmgkb.org/ (accessed May 19, 2022).

14 https://www.genecards.org/ (accessed May 19, 2022).

15 http://www.bioinformatics.com.cn/ (accessed May 20, 2022).

16 https://cn.string-db.org/ (accessed June 1, 2022).

17 https://david.ncifcrf.gov/ (accessed June 10, 2022).

18 http://www.alzdata.org/ (accessed June 11, 2022).

19 https://pubchem.ncbi.nlm.nih.gov/ (accessed June 13, 2022).

20
https://www.rcsb.org/

21 http://pantherdb.org/ (accessed June 15, 2020).

## Supplementary material

Supplementary material The ^1^H NMR of the active ingredients extracted and isolated from *Fibraurea recisa Pierre* can be seen in Supplementary Figures 1–5. The physicochemical properties of the main ingredients of FRP can be seen in Supplementary Table 1. The top 20 effective pathways to AD with the main ingredients of FRP can be seen in Supplementary Table 2.
