# Supplementary material for: Potential bioactive compounds and mechanisms of Fibraurea recisa Pierre for the treatment of Alzheimer’s disease analyzed by network pharmacology and molecular docking prediction
Source: Front Aging Neurosci. 2022 Dec 8;14:1052249. doi: 10.3389/fnagi.2022.1052249 (PMC9772884; doi:10.3389/fnagi.2022.1052249)
Supplement: Supplementary material — The 1H NMR of the active ingredients extracted and isolated from Fibraurea recisa Pierre can be seen in Supplementary Figures 1–5. The physicochemical properties of the main ingredients of FRP can be seen in Supplementary Table 1. The top 20 effective pathways to AD with the main ingredients of FRP can be seen in Supplementary Table 2. [file Data_Sheet_1.PDF]

Supplementary Materials

Potential bioactive compounds and mechanisms of *Fibraurea recisa Pierre* for the treatment of Alzheimer's disease analyzed by network pharmacology and molecular docking prediction

Shishuai Wang[1,2,5], Yixuan Ma[1,2,5], Yuping Huang[1]; Yuhui Hu[6*], Yushan Huang[1*], Yi Wu[2, 3*],

[1]Center for Evidence Based Medical and Clinical Research, First Affiliated Hospital of Gannan Medical University, Ganzhou 341000, China

[2]Key Laboratory of Prevention and treatment of cardiovascular and cerebrovascular diseases, Ministry of Education, Gannan Medical University, Ganzhou 341000, China.

[3]Jiangxi Province Key Laboratory of Biomaterials and Biofabrication for Tissue Engineering, Gannan Medical University, Ganzhou 341000, China.

[4]Department of Biochemistry and Molecular Biology, Gannan Medical University, Ganzhou 341000, China

[5]College of Pharmacy, Gannan Medical University, Ganzhou 341000, China

[6]Medical College, Jinggangshan University, Ji'an, Jiangxi 343009, China

*** Correspondence:**

Corresponding Author: Yi Wu, wuyi@gmu.edu.cn.com; Yushan Huang,3218680@qq.com;Yuhui Hu,9919940052@jgsu.edu.cn

^{1}H-NMR of the extract of the Chinese medicine *Fibraurea recisa Pierr*

Supporting data	Pages
Fig. S1. ^{1}H-NMR (DMSO) spectrum of sitogluside	2
Fig. S2. ^{1}H-NMR ($CDCl_3$) spectrum of β-sitosterol	2
Fig. S3. ^{1}H-NMR (DMSO) spectrum of jatrorrhizine	3
Fig. S4. ^{1}H-NMR (DMSO) spectrum of palmatine	3
Fig. S5. ^{1}H-NMR (DMSO) spectrum of berberine	4
Tab. S1. Physicochemical properties of the main ingredients of FRP	4
Tab. S2. Top 20 effective pathways to AD with the main ingredients of FRP	5

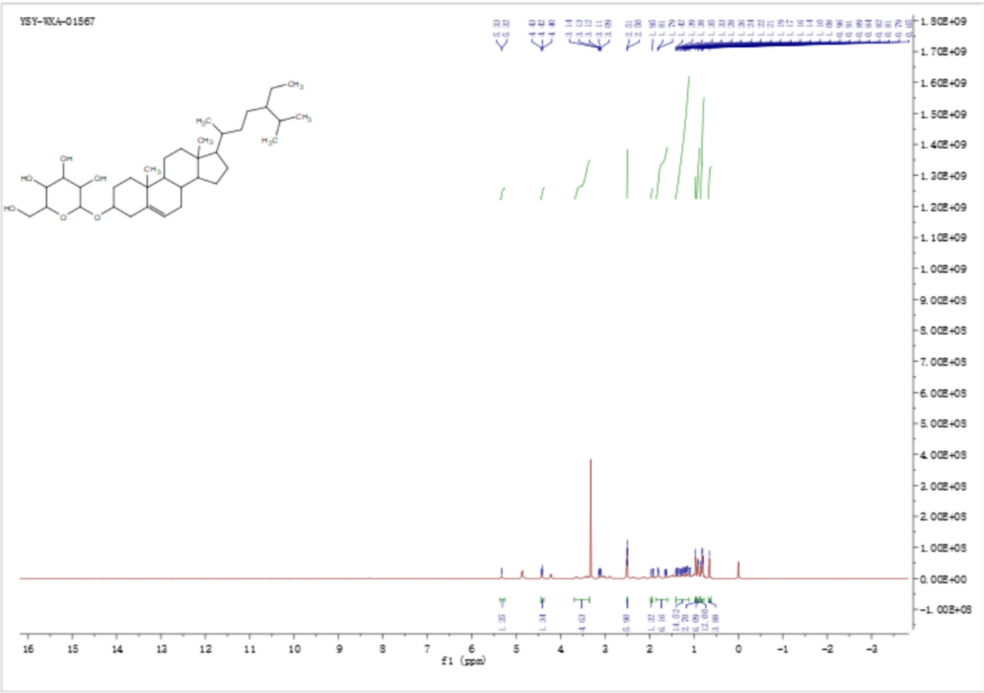

Figure 1. ^{1}H-NMR (DMSO) spectrum of sitogluside

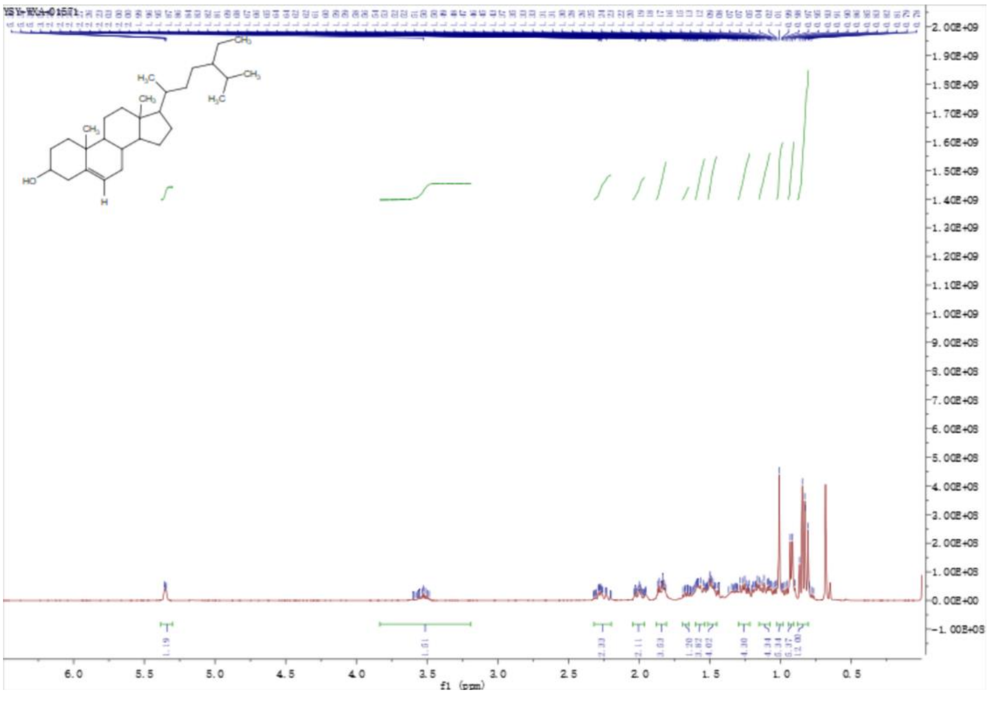

Fig. S2. ^{1}H-NMR ($CDCl_3$) spectrum of β -sitosterol

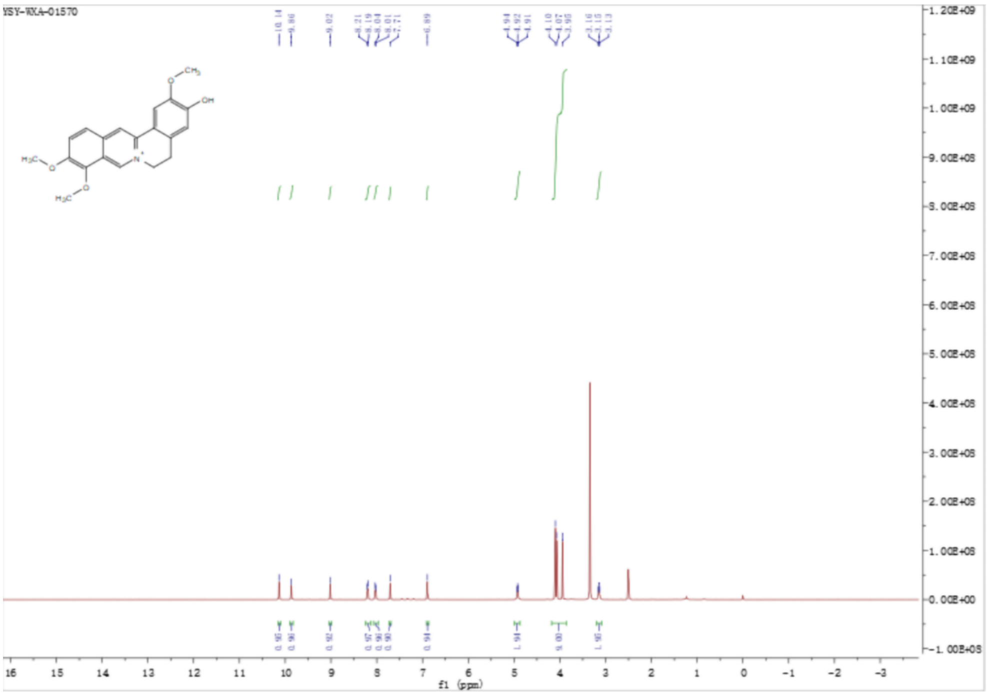

Fig. S3. ^{1}H-NMR (DMSO) spectrum of jatrorrhizine

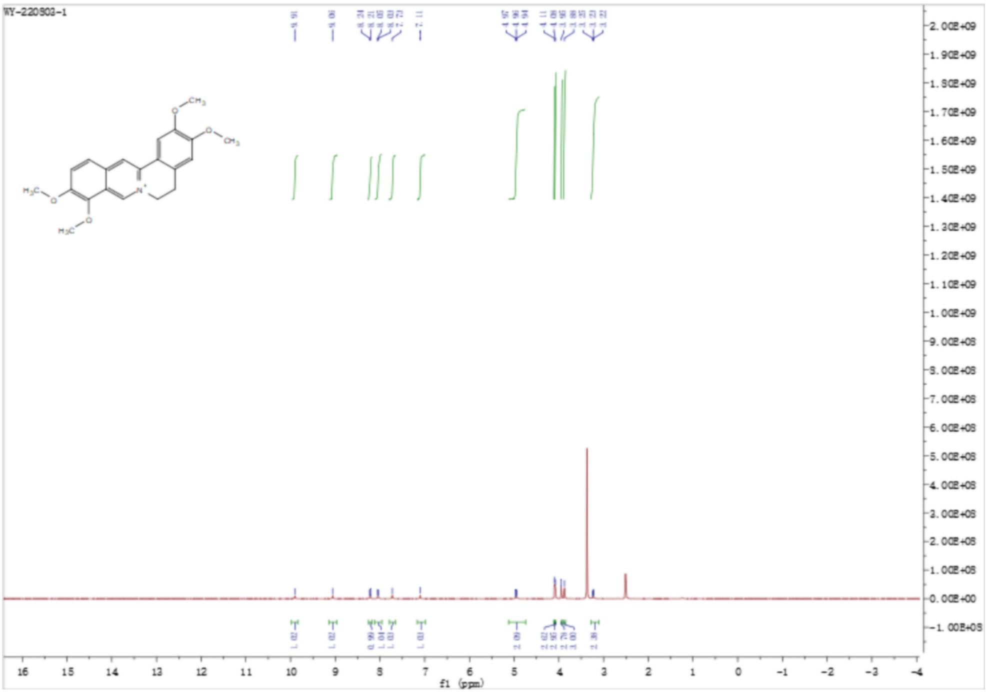

Fig. S4. ^{1}H-NMR (DMSO) spectrum of palmatine

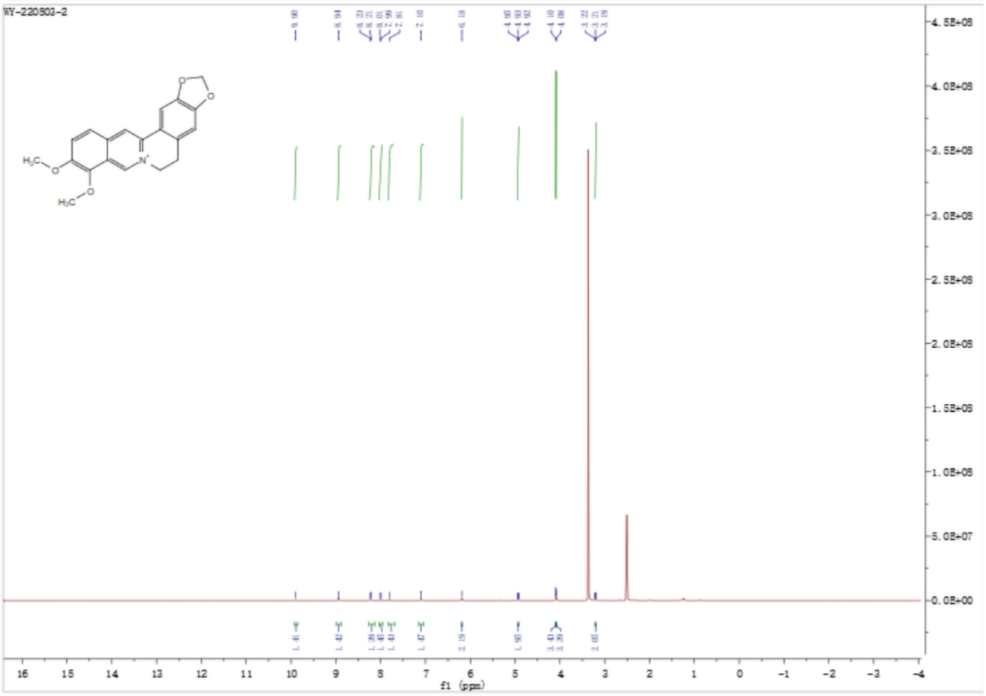

Fig. S5. ^{1}H-NMR (DMSO) spectrum of berberine

NO.	Compounds	MW<br><500	HBA<br><10	HBD<br>≤5	LogP<br>≤4.15	LopS	Rbon	BBB	TPSA(Å2)	Sascore	Lipinski's Violations	Biavailability Score
		Lipinski Rules										
1	Palmatine	352.150	4	0	3.785	−5.366	4	0.923	40.8	2.62	0	0.55
2	Jatrorrhizine	338.140	4	1	3.526	−5.308	3	0.787	51.8	2.74	0	0.55
3	Berberine	336.120	4	0	4.309	−6.311	2	0.914	40.8	2.53	0	0.55
4	Tetrahydropalmatine	355.180	5	0	2.653	−2.443	4	0.988	40.16	3.08	0	0.55
5	Triptolide	360.16	6	1	2.817	−3.901	1	0.616	84.12	1.7	0	0.55
6	Corydalmine	341.160	5	1	2.476	−1.832	3	0.992	51.16	2.71	0	0.55
7	Columbamine	338.14	4	1	3.467	−5.362		0.778	51.8	2.33	0	0.55
8	Roemerine	279.13	3	0	3.589	−2.789	0	0.988	21.7	3.13	0	0.55
9	Oleanolicacid	456.36	3	2	6.486	−4.086	1	0.694	57.530	6.06	1	0.85
10	Nerolidol	222.200	1	1	5.179	−3.897	7	0.175	20.230	4.19	0	0.55
11	Sitogluside	576.440	6	4	5.97	−4.432	9	0.059	99.380	5.51	1	0.55
12	β-Sitosterol	414.39	1	1	7.663	−7.052	6	0.84	20.23	4.38	1	0.55

Tab. S1 The physicochemical properties of the main ingredients of FRP

MW: molecular weight. HBA: hydrogen bond acceptor. HBD: hydrogen bond donor. LogP: Log of the octanol/water partition coefficient. LopS: Log of the aqueous solubility. Rbon: rotatable bonds. BBB: Blood–Brain Barrier TPSA: Topological Polar Surface Area. SAscore: Synthetic accessibility score BBB: Blood–Brain Barrier TPSA: Topological Polar Surface Area. SAscore: Synthetic accessibility score.

Term	Pathway	Count	PValue	Genes	Fold Enrichment
hsa04080	Neuroactive ligand-receptor interaction	51	2.72E-22	CHRM2, CHRM3, THRB, CHRM1, CHRM4, CHRM5, HTR2B, HTR2C, ADRA1D, HTR2A, ADRA1B, HTR4, NR3C1, ADRA1A, HTR6, HTR7, TSPO, OPRM1, ADRA2C, F2, ADRA2B, ADRA2A, MTNR1A, ADORA2A, AGTR1, AGTR2, GRIA1, OPRD1, CHRNA4, PTGER2, CHRNA7, ADRB1, PLG, ADRB2, GLRA1, CNR2, HRH2, CNR1, DRD1, DRD2, DRD3, DRD4, DRD5, HTR1E, HTR1D, HTR1A, HTR1B, OPRK1, TRPV1, HTR5A, P2RX7	5.084290813
hsa04151	PI3K-Akt signaling pathway	38	1.31E-12	CHRM2, CSF1R, GSK3B, CHRM1, PIK3CD, PIK3R1, FGF1, FGF2, EGFR, PIK3CG, IGF1R, IKBKB, KDR, AKT1, MAPK1, RAC1, JAK2, MCL1, MAPK3, PDGFRB, NTRK1, MAP2K1, HSP90AA1, SYK, IL2, MTOR, VEGFA, CREB1, CDK6, RPS6KB1, CDK4, KIT, CDK2, MDM2, RAF1, MET, FGFR1, EPHA2	3.873906305
hsa04020	Calcium signaling pathway	37	2.61E-17	RET, CHRM2, CHRM3, CHRM1, CHRNA7, CHRM5, HTR2B, HTR2C, ADRA1D, ADRB1, ADRB2, HTR2A, FGF1, ADRA1B, HTR4, FGF2, ADRA1A, EGFR, MYLK, HTR6, HTR7, HRH2, KDR, DRD1, PLCG1, PRKACA, DRD5, PDGFRB, NTRK1, NOS2, HTR5A, VEGFA, P2RX7, ADORA2A, AGTR1, MET, FGFR1	5.563643068
hsa04024	cAMP signaling pathway	35	9.86E-17	GRIA1, CHRM2, CHRM1, PTGER2, PIK3CD, ADRB1, ADRB2, PIK3R1, HTR4, SLC9A1, MAPK8, HTR6, AKT1, MAPK1, EP300, DRD1, RAC1, DRD2, PRKACA, DRD5, MAPK3, JUN, MAP2K1, HTR1E, PDE4D, HTR1D, HTR1A, HTR1B, BRAF, MAPK10, CREB1, ADORA2A, RAF1, PPARA, CFTR	5.715372602
hsa04010	MAPK signaling pathway	33	1.76E-11	CSF1R, FGF1, FGF2, TNF, EGFR, IGF1R, IKBKB, CDC42, MAPK8, CASP3, KDR, AKT1, MAPK1, RAC1, PRKACA, MAPK3, PDGFRB, NTRK1, JUN, MAP2K1, BRAF, IRAK4, MAPK14, TGFBR1, TGFBR2, VEGFA, MAPK10, IL1B, KIT, RAF1, MET, FGFR1, EPHA2	4.050749503
hsa04014	Ras signaling pathway	31	1.32E-12	CSF1R, PIK3CD, PIK3R1, FGF1, FGF2, EGFR, IGF1R, IKBKB, CDC42, MAPK8, HTR7, KDR, ABL1, AKT1, MAPK1, PLCG1, RAC1, PRKACA, MAPK3, PDGFRB, NTRK1, MAP2K1, PTPN11, VEGFA, MAPK10, ZAP70, KIT, RAF1, MET, FGFR1, EPHA2	4.760610055
hsa04726	Serotonergic synapse	29	2.11E-19	APP, MAOB, MAOA, HTR2B, HTR2C, HTR2A, CYP2C19, HTR4, PTGS2, SLC6A4, PTGS1, HTR6, HTR7, CYP2D6, CASP3, ALOX5, MAPK1, PRKACA, SLC18A2, MAPK3, MAP2K1, HTR1E, HTR1D, HTR1A, HTR1B, HTR3A, BRAF, HTR5A, RAF1	9.100577145
hsa05022	Pathways of neurodegeneration - multiple diseases	29	1.10E-04	GRIA1, APP, CHRM3, GSK3B, CHRM1, CHRNA7, LRRK2, CHRM5, PTGS2, TNF, SLC6A3, CASP7, MAPK8, TUBB3, CASP3, MAPK1, PLCG1, RAC1, MAPK3, MAP2K1, NOS2, CSNK2A1, SIGMAR1, BRAF, MAPK14, MTOR, MAPK10, IL1B, RAF1	2.198668848
hsa04015	Rap1 signaling pathway	28	1.63E-11	CSF1R, SRC, PIK3CD, PIK3R1, FGF1, FGF2, EGFR, IGF1R, CDC42, CNR1, KDR, AKT1, MAPK1, PLCG1, RAC1, DRD2, MAPK3, PDGFRB, MAP2K1, BRAF, MAPK14, VEGFA, ADORA2A, KIT, RAF1, MET, FGFR1, EPHA2	4.81179941
hsa05010	Alzheimer disease	28	6.43E-06	APP, CHRM3, GSK3B, CHRM1, CHRNA7, CHRM5, PIK3CD, PIK3R1, PTGS2, TNF, IKBKB, CASP7, MAPK8, TUBB3, CASP3, AKT1, MAPK1, MAPK3, MAP2K1, NOS2, CSNK2A1, ADAM10, BRAF, MTOR, BACE1, MAPK10, IL1B, RAF1	2.631452802
hsa04933	AGE-RAGE signaling pathway in diabetic complications	27	7.38E-19	PIK3CD, PIK3R1, TNF, ICAM1, CDC42, MAPK8, CASP3, AKT1, MAPK1, PLCG1, RAC1, JAK2, MAPK3, JUN, PRKCE, MMP2, STAT3, MAPK14, SELE, F3, TGFBR1, TGFBR2, VEGFA, MAPK10, CDK4, IL1B, AGTR1	9.743893805
hsa04926	Relaxin signaling pathway	23	4.76E-12	MAP2K1, JUN, NOS2, MMP1, SRC, MMP2, PIK3CD, PIK3R1, MAPK14, MMP9, EGFR, TGFBR1, TGFBR2, VEGFA, MAPK10, MAPK8, MMP13, CREB1, AKT1, MAPK1, RAF1, PRKACA, MAPK3	6.434382932
hsa05235	PD-L1 expression and PD-1 checkpoint pathway in cancer	22	1.76E-14	MAP2K1, JUN, CSNK2A1, STAT3, PIK3CD, PTPN11, PIK3R1, MAPK14, EGFR, MTOR, IKBKB, ZAP70, CD4, RPS6KB1, LCK, TLR9, AKT1, MAPK1, PLCG1, JAK2, RAF1, MAPK3	8.920751715
hsa04660	T cell receptor signaling pathway	22	4.79E-13	GSK3B, MAP2K1, JUN, PIK3CD, PIK3R1, MAPK14, TNF, IL2, MAPK10, CDC42, IKBKB, ZAP70, CD4, MAPK8, PTPRC, CDK4, LCK, AKT1, MAPK1, PLCG1, RAF1, MAPK3	7.634104833
hsa04668	TNF signaling pathway	22	2.21E-12	MAP2K1, JUN, MMP3, PIK3CD, PIK3R1, PTGS2, MAPK14, SELE, TNF, MMP9, ICAM1, MAPK10, IKBKB, MMP14, CASP7, MAPK8, CREB1, IL1B, CASP3, AKT1, MAPK1, MAPK3	7.088811631
hsa04380	Osteoclast differentiation	22	3.22E-11	CSF1R, MAP2K1, JUN, SYK, PIK3CD, PIK3R1, MAPK14, TNF, TGFBR1, TGFBR2, MAPK10, IKBKB, MAPK8, CREB1, LCK, IL1B, BTK, AKT1, MAPK1, PPARG, RAC1, MAPK3	6.202710177
hsa04931	Insulin resistance	21	9.63E-12	PTPN1, GSK3B, PRKCE, NR1H2, STAT3, PIK3CD, NR1H3, PTPN11, PYGL, PIK3R1, TNF, PTPRF, MTOR, MAPK10, IKBKB, MAPK8, CREB1, RPS6KB1, AKT1, PPARA, OGT	7.017207473
hsa04722	Neurotrophin signaling pathway	21	6.15E-11	NTRK1, GSK3B, MAP2K1, JUN, PIK3CD, BRAF, PTPN11, PIK3R1, IRAK4, MAPK14, MAPK10, CDC42, IKBKB, MAPK8, ABL1, AKT1, MAPK1, PLCG1, RAC1, RAF1, MAPK3	6.368558043
hsa04659	Th17 cell differentiation	20	8.30E-11	JUN, HSP90AA1, STAT3, AHR, MAPK14, TGFBR1, IL2, MTOR, TGFBR2, MAPK10, IKBKB, ZAP70, CD4, MAPK8, LCK, IL1B, MAPK1, PLCG1, JAK2, MAPK3	6.683054736
hsa04068	FoxO signaling pathway	20	2.53E-09	MAP2K1, STAT3, PIK3CD, BRAF, PIK3R1, MAPK14, EGFR, TGFBR1, TGFBR2, IGF1R, MAPK10, IKBKB, MAPK8, CDK2, MDM2, AKT1, EP300. MAPK1. RAF1. MAPK3	5.509693981

Tab. S2 Top 20 effective pathways to AD with the main ingredients of FRP
